# Functional heterogeneity of TP53 mutants in venetoclax-resistant AML: mechanisms, clinical implications, and therapeutic opportunities

**DOI:** 10.3389/fonc.2026.1921345

**Published:** 2026-07-22

**Authors:** Maierhaba Maimaitituerhong, Jinling Xiao, Wenji Gao, Ruiqi Zheng, Yage Fan, Rong Zhang, Yi Wang, Fengmei Deng, Leiming Xia

**Affiliations:** 1 School of Basic Medical Sciences, Xinjiang Second Medical College, Karamay, China; 2 Department of Oncology, Hefei first people`s hospital, Hefei, China; 3 School of Basic Medical Sciences, Chengdu Medical College, Chengdu, China

**Keywords:** acute myeloid leukemia (AML), functional heterogeneity, leukemia stem cell (LSC), metabolic reprogramming, precision medicine, TP53 mutation, venetoclax resistance

## Abstract

TP53 mutations are strongly associated with resistance to venetoclax in acute myeloid leukemia (AML) and represent a major challenge in current treatment strategies. Importantly, different TP53 mutants exhibit substantial functional heterogeneity, leading to distinct resistance phenotypes that are not adequately captured by conventional variant allele frequency (VAF)-based stratification. Emerging evidence suggests that TP53 mutations promote venetoclax resistance through multiple mechanisms, including apoptotic dysregulation, metabolic reprogramming, enhancement of leukemic stem cell properties, and epigenetic remodeling, with the relative contribution of each pathway varying among mutant types. This review systematically summarizes recent advances in the molecular mechanisms underlying TP53-mediated venetoclax resistance, with a particular focus on how mutant-specific functional differences shape therapeutic responses. Unlike previous broad reviews of TP53-mutated AML, this article specifically addresses venetoclax resistance as a clinically critical therapeutic challenge. We further discuss the limitations of current VAF-based classification systems and propose a practical framework that integrates mutant-specific resistance biology with precision therapeutic strategies.

## 1 Introduction

### 1.1 TP53-mutated AML represents a major challenge to venetoclax-based therapy

TP53 mutations, which carry significant clinical implications in acute myeloid leukemia (AML), occur in approximately 10%-20% of AML patients and are more frequently observed in those with adverse cytogenetic features such as complex karyotype or deletion of chromosome 7 (1). TP53 mutation is a well-established poor prognostic factor in AML (2), manifesting in several clinical challenges: First, patients with TP53 mutations often experience unfavorable outcomes, showing significantly reduced complete response rates to standard induction chemotherapy, increased risk of relapse, and markedly shorter overall survival (OS) (3). Second, TP53 mutation confers intrinsic multi-drug resistance to various conventional chemotherapeutic agents (4). Finally, TP53 mutation also limits therapeutic efficacy in the era of molecular targeted therapy by frequently causing primary or secondary resistance to agents including the BCL-2 inhibitor venetoclax, severely restricting its clinical application and effectiveness, with currently no effective interventions to address this issue (5).

### 1.2 TP53 mutations should not be viewed as a homogeneous molecular entity

Venetoclax is a BH3-mimetic agent that specifically inhibits BCL-2, inducing mitochondrial-dependent apoptosis in leukemia cells (6). However, its efficacy is significantly inferior in TP53-mutated AML compared to wild-type patients (7). Studies indicate a resistance rate of approximately 60% to venetoclax-based regimens in this AML population, severely limiting its clinical utility (5, 8, 9). Specifically, approximately 30% of TP53-mutated AML patients exhibit primary resistance, and almost all initial responders eventually relapse, indicating acquired resistance (10). These clinical observations underscore the urgent need to elucidate the mechanisms of venetoclax resistance in TP53-mutated AML and to develop effective strategies resistance to overcome. Although mutant p53 does not directly regulate BCL2 family genes, it controls a broad pro-apoptotic network (11)—including the transcriptional activation of BAX and PUMA and suppression of BCL-xL—that is essential for venetoclax sensitivity. Additionally, p53 can directly bind to BCL-2 at the mitochondrial membrane via its DNA-binding domain (12), competitively displacing pro-apoptotic proteins such as BAX and facilitating mitochondrial outer membrane permeabilization (MOMP) (13). These dual mechanisms—both transcriptional and non-transcriptional—position p53 as a central determinant of venetoclax response, and their disruption in TP53-mutated AML provides a rational foundation for developing therapeutic strategies aimed at restoring p53-associated apoptotic function.

### 1.3 Functional heterogeneity of TP53 mutants may drive distinct venetoclax resistance phenotypes

This review comprehensively elaborates on the molecular mechanisms underlying venetoclax resistance in TP53-mutated AML, aiming to explore the potential association between the functional heterogeneity of different TP53 point mutations and venetoclax resistance, and to reveal the potential limitations of current risk stratification and therapeutic strategies. Regarding scientific value, this review contributes in the following aspects: First, by integrating recent advances in the structural and functional heterogeneity of TP53 mutations, it provides a conceptual framework for understanding how different mutants drive differential resistance phenotypes. Second, it synthesizes key molecular events across major resistance pathways—including apoptotic pathway inactivation, metabolic reprogramming, cancer stem cell maintenance, and epigenetic dysregulation—thereby identifying potential nodes for therapeutic intervention. Third, it advances a precision medicine paradigm based on functional classification of TP53 mutants, moving beyond the current risk stratification centered on biallelic mutation status and variant allele frequency (VAF), and establishes a direct link between TP53 mutant function and venetoclax sensitivity to inform individualized treatment decisions. Of course, given the limited depth of current research on the biological functions of the TP53 point mutation spectrum, directly elucidating the specific contributions of individual point mutations to venetoclax resistance remains premature; nevertheless, this direction is undoubtedly worthy of further exploration. Collectively, emerging evidence suggests that venetoclax resistance in TP53-mutated AML should no longer be viewed as a uniform biological phenotype, but rather as a spectrum of mutant-specific adaptive states driven by distinct structural and functional properties of individual TP53 variants.

### 1.4 Scope and conceptual framework of this review

**Figure 1:**
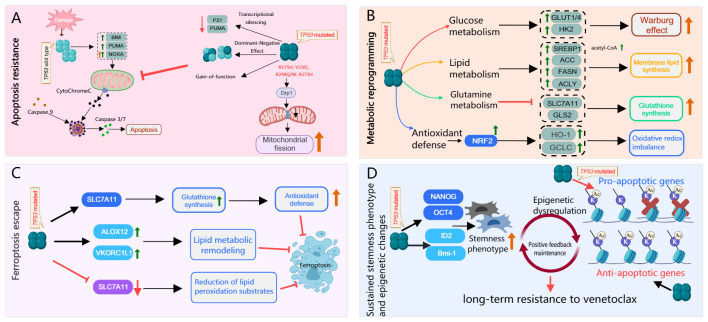
Functional heterogeneity of TP53 mutants drives venetoclax resistance in acute myeloid leukemia. **(A)** Under stresses such as DNA damage, wild-type p53 mediates cell cycle arrest and mitochondrial apoptosis through activation of p21 and PUMA/NOXA, respectively. TP53 loss-of-function mutations abolish the transcriptional activation of p21 and PUMA, dominant-negative mutations suppress residual wild-type p53 activity, while gain-of-function mutations (e.g., R175H, R248Q) stabilize anti-apoptotic proteins and induce excessive mitochondrial fission and mitophagy dysregulation via DRP1 activation. Together, these mechanisms block apoptotic pathways, rendering cells resistant to venetoclax. **(B)** Mutant p53 drives metabolic reprogramming by relieving inhibition of GLUT1/4 and HK2 while enhancing their expression to accelerate the Warburg effect; upregulating ACC, FASN, and ACLY to promote fatty acid synthesis; and disrupting GLS2 activation while upregulating SLC7A11 to increase glutamine utilization and glutathione production, thereby providing biosynthetic modules and energy sources for drug-resistant cells. **(C)** Mutant p53 establishes a robust antioxidant barrier by overactivating the NRF2-mediated HO-1 and GCLC antioxidant pathways, combined with upregulation of SLC7A11, inhibition of PLTP, and activation of ALOX12, which synergistically reduce lipid peroxidation and enable ferroptosis evasion to maintain cell survival. **(D)** Structural or contact mutations in TP53 disrupt the DNA-binding domain, impairing recruitment of p300/CBP and HDACs. This leads to hypoacetylation and silencing of pro-apoptotic genes, alongside hyperacetylation and aberrant expression of anti-apoptotic genes and drug efflux pumps, establishing a pro-survival transcriptional permissive state. Concurrently, relief of NANOG and OCT4 suppression, together with upregulation of ID2 and Bmi-1, promotes leukemia stem cell expansion, which sustains venetoclax tolerance through fatty acid oxidation and MCL-1 upregulation.

This review focuses on the functional heterogeneity of TP53 mutants in venetoclax-resistant AML. We summarize how distinct TP53 mutation types differentially influence apoptotic signaling, mitochondrial regulation, metabolic adaptation, stem cell maintenance, and epigenetic remodeling. We further discuss the limitations of current VAF-based stratification systems and propose a conceptual framework for mutant function-guided precision therapeutic strategies (**Figure 1**).

## 2 Molecular features and functional heterogeneity of TP53 mutants

### 2.1 TP53 mutation types and their functional heterogeneity in venetoclax resistance

TP53 mutations in AML are predominantly missense mutations (~75%) clustered within the DNA-binding domain (DBD) (14), with six recurrent hotspots including R175H, G245S, R248Q/W, R273H, and R282W (15, 16). The remaining mutations mainly consist of nonsense and frameshift variants that result in premature protein truncation (17, 18). These distinct mutation classes exert fundamentally different effects on p53 function and may therefore contribute differently to venetoclax resistance. A systematic p53 mutation library further supports this concept by quantitatively assessing thousands of DNA-binding domain variants in human cells. This study showed that p53 mutations can be broadly separated into variants that retain wild-type-like function and those that disrupt p53 activity, while also revealing substantial heterogeneity even among hotspot mutations (17). These findings indicate that TP53 mutation status alone is insufficient to infer functional consequence.

Loss of function (LOF) represents the most common consequence of TP53 mutation. Structural disruption of the DBD impairs sequence-specific DNA binding and compromises the transcriptional activation of critical tumor suppressor pathways, including apoptosis, cell-cycle arrest, and DNA damage responses (19, 20). Given the dependence of venetoclax-induced apoptosis on intact p53-mediated apoptotic priming, LOF is widely considered a major contributor to venetoclax resistance.

Dominant-negative effects (DNE) constitute an additional mechanism that is particularly relevant to missense mutations. Unlike truncating variants, many DBD missense mutants retain the ability to oligomerize with wild-type p53 through tetramer formation, thereby suppressing the transcriptional activity of the remaining functional allele. Functional studies, including saturation mutagenesis and hematopoietic models, have demonstrated that DBD missense mutants frequently exhibit DNE and can confer a selective advantage under conditions of genotoxic stress (21). Importantly, the strength of DNE varies considerably among different TP53 mutants, further contributing to the functional heterogeneity observed in TP53-mutated AML (17, 22). While DNE has been implicated in clonal evolution and therapeutic resistance, its precise contribution to venetoclax resistance remains incompletely understood.

In contrast, gain-of-function (GOF) mutations acquire novel oncogenic activities beyond simple loss of tumor suppressor function. Through altered protein–protein interactions and transcriptional programs, GOF mutants may promote survival signaling (23), metabolic adaptation, and therapy resistance. However, compared with LOF and DNE, direct evidence linking specific GOF activities to venetoclax resistance remains limited. Current evidence therefore supports LOF and DNE as the primary mechanisms underlying TP53-associated venetoclax resistance, whereas the role of GOF requires further investigation.

Collectively, the diverse functional consequences of TP53 mutations—including LOF, DNE, and potentially GOF—suggest that TP53-mutated AML should not be viewed as a biologically homogeneous entity (14, 24). Instead, distinct mutation types may generate different resistance phenotypes and therapeutic vulnerabilities, providing a rationale for function-guided classification and precision therapeutic strategies.

### 2.2 DBD mutations and p53-DNA binding ability

The DBD requires zinc coordination (Cys176, His179, Cys238, and Cys242) for structural stability (25). Mutations in this region can impair DNA binding by altering contact residues, disrupting zinc coordination, or inducing conformational changes (26). While these structural defects are well-established in p53 dysfunction, their direct link to venetoclax resistance remains inferential, based on the essential role of p53-dependent pro-apoptotic transcription in BCL-2 inhibitor sensitivity.

A recent computational study of 148 DBD missense mutations identified two distinct structural mechanisms (27): some mutations (e.g., R248P, N239S) reduce DNA-binding affinity, while others (e.g., C238Y, P278R) enhance affinity but compromise structural stability. Notably, certain mutations (e.g., E285A, M243T) maintained stability with enhanced DNA binding, suggesting potential “rescuing” effects.

These structural insights offer a hypothesis-generating framework for venetoclax resistance: mutations severely impairing DNA binding likely compromise BH3-only protein activation, raising the apoptotic threshold and potentially diminishing venetoclax efficacy (28). Conversely, mutations preserving DNA binding might retain partial p53 function, potentially conferring differential drug sensitivity (17, 29). However, direct evidence linking specific DBD mutation types to venetoclax response remains limited, and these hypotheses require systematic validation (17).

### 2.3 Disruption of protein–protein interaction networks

Beyond impaired DNA binding, TP53 mutations may alter critical protein–protein interaction networks. Among these, sequestration of p63 and p73 by mutant p53 is the best-characterized mechanism, leading to impaired apoptotic signaling and enhanced survival (30). Because p63/p73 contribute to the transcriptional regulation of several pro-apoptotic genes, their inactivation may further elevate the apoptotic threshold and reduce sensitivity to venetoclax (31).

In addition, several hotspot mutants, including R175H, R248Q, R273H, and Y220C, can be stabilized through interactions with molecular chaperones such as HSP90 (32), thereby prolonging mutant p53 accumulation and sustaining dominant-negative or gain-of-function activities.

However, direct evidence linking specific PPI alterations to venetoclax resistance in AML remains limited. Most current observations derive from other cancer types, and their relevance to TP53-mutated AML requires further validation.

### 2.4 Metabolic reprogramming

TP53 mutations reprogram cellular metabolism to raise the apoptotic threshold and diminish venetoclax sensitivity. Loss of p53-mediated suppression of GLUT1/4 and HK2 drives aerobic glycolysis (33, 34), while depression of SLC7A11 enhances glutathione synthesis and antioxidant capacity, neutralizing venetoclax-induced oxidative stress (35). Concurrently, upregulated fatty acid oxidation sustains oxidative phosphorylation independently of BCL-2, providing an alternative energy route that bypasses the drug’s primary target (36, 37). These metabolic adaptations collectively establish a stress-resistant cellular milieu that attenuates the pro-apoptotic impact of BCL-2 inhibition (38).

### 2.5 Leukemia stem cell maintenance

TP53 mutations expand the leukemic stem cell (LSC) reservoir (39), creating a durable source of venetoclax-resistant cells. By relieving p53-dependent suppression of NANOG and OCT4 (40), mutant p53 enhances LSC self-renewal and impedes differentiation (41). Although LSCs are intrinsically dependent on BCL-2–regulated oxidative phosphorylation—a vulnerability targeted by venetoclax (42)—TP53-mutated LSCs escape through compensatory MCL-1 upregulation (via the METTL3–MYC–MCL1 axis) (43) and enhanced fatty acid oxidation (36). This dual defect, combining impaired apoptotic priming with selective enrichment of a drug-tolerant stem cell population (38), positions LSCs as a central hub of venetoclax resistance in TP53-mutated AML.

### 2.6 Epigenetic dysregulation

TP53 mutations disrupt the epigenetic machinery that licenses apoptotic gene expression, locking cells in a transcriptionally resistant state (11). Wild-type p53 recruits p300/CBP to acetylate and activate pro-apoptotic promoters (PUMA, BAX) and engages HDACs to silence anti-apoptotic genes such as BCL-2 and survivin (44, 45). Mutant p53 loses this bidirectional control, resulting in hypoacetylation and silencing of pro-apoptotic targets alongside aberrant activation of survival programs (29). This histone-level dysregulation—distinct from the DNA methylation defects targeted by hypomethylating agents—may explain why HMA–venetoclax combinations fail to overcome resistance in TP53-mutated AML (Section 4.1) (42), and suggests that HDAC inhibitors or p300/CBP modulators could restore venetoclax sensitivity) (46).

The functional heterogeneity detailed above—spanning apoptotic priming defects, metabolic adaptation, LSC expansion, and epigenetic rewiring—generates a spectrum of mutant-specific resistance phenotypes that cannot be captured by mutation burden alone (47). Current clinical stratification, however, relies predominantly on variant allele frequency (VAF), which measures clone abundance without regard to mutation type or functional consequence (48). The following section examines why this VAF-centric framework is theoretically inadequate for predicting venetoclax response in TP53-mutated AML.

## 3 Current evaluation system based on VAF has theoretical limitations in predicting venetoclax response

The current evaluation system centered on variant allele frequency (VAF) has profound theoretical limitations, as it fails to capture the biological complexity and functional heterogeneity of TP53 mutations.

### 3.1 VAF cannot capture functional heterogeneity or clonal dynamics

Current clinical practice relies heavily on VAF for prognostic assessment and efficacy prediction, with VAF ≥40% commonly defined as high risk (49). This approach has two major limitations.

First, VAF ignores functional heterogeneity. It reflects only mutant clone abundance, not the actual impact of different mutant types on p53 pathway activity (50). For instance, DBD missense mutations (e.g., R248W) can potently inhibit wild-type p53 through dominant-negative effects, whereas certain nonsense mutations outside the DNA-binding domain may cause only simple loss of function (21). These functional differences—which have direct implications for venetoclax sensitivity—are invisible to VAF. This limitation is conceptually supported by large-scale functional profiling of p53 variants, which demonstrated that mutation prevalence or allele abundance does not fully capture mutation-specific functional effects (51). Some variants may be relatively rare but strongly disruptive, whereas others may retain partial wild-type-like activity despite being classified simply as TP53-mutated. Therefore, a VAF-centered evaluation system cannot distinguish between biologically distinct TP53 mutant states (47).

Second, VAF fails to capture clonal dynamic evolution. Conventional VAF assessment cannot track the adaptive changes of TP53-mutant clones under therapeutic pressure (52). Studies have shown that even after achieving CR with venetoclax-based combination therapy, TP53-mutant clones may re-expand following a transient decline in VAF, suggesting the latency and evolution of resistant subclones (53).

### 3.2 Heterogeneity of TP53 mutations confers distinct venetoclax resistance phenotypes

Resistance to venetoclax depends not only on mutant clone abundance but more critically on the functional subtype of the mutant and its differential impact on apoptotic pathways (50).

In the BAX/BAK apoptotic axis, DBD mutations can severely impair p53-mediated transcriptional activation of BAX, weakening mitochondrial apoptotic initiation (54). In contrast, certain Non-DBD mutations may cause only mild BAX downregulation, with more limited impact on apoptosis (47). This gradient of apoptotic inhibition, resulting from functional differences in mutation sites, cannot be captured by VAF (47).

Similarly, mitochondrial dynamics are differentially affected by distinct TP53 mutants. Some mutations lead to complete loss of DRP1 inhibitory function, while others regulate through non-transcriptional mechanisms (55). These functional heterogeneity alters mitochondrial membrane potential and apoptotic threshold, directly influencing venetoclax efficacy, yet remain indistinguishable by VAF alone (53).

In summary, different functional types of TP53 mutants may mediate distinct resistance phenotypes even at identical VAF levels. The current VAF-dependent evaluation system obscures these critical biological differences and cannot accurately predict venetoclax response (50).

### 3.3 VAF cannot predict mutant “druggability” for p53 reactivators

p53 reactivators (e.g., APR-246, NSC59984) offer theoretical promise for TP53-mutated AML, but their clinical efficacy is constrained by mutant-specific functional defects that VAF cannot capture (56). The key issue is mutant “druggability”, whether a given mutant possesses structural features amenable to pharmacological restoration (57).

Conformational restorability determines drug sensitivity. Different mutants exhibit significant spatial conformations that affect reactivators binding (58). The Y220C mutation creates a targetable hydrophobic pocket that can be stabilized by small molecules, partially restoring transcriptional activity (57). In contrast, core DBD mutations like R175H cause global structural collapse, rendering them “structurally silent” to current reactivators (47). VAF cannot identify these “drug-rescuable” from “non-rescuable” mutations.

Transcriptional-independent functional loss further limits synergy. Even when transcriptional activity is partial restored, some mutants may lack p53’s transcription-independent pro-apoptosis function, such as direct interaction with Bcl-2 family proteins or regulation of mitochondrial membrane potential (59). This “functional gap” impedes synergy with venetoclax, especially when apoptotic threshold is already elevated. Again, VAF provides no insight into this critical defect (50).

Therefore, high VAF does not predict reactivators efficacy, and low VAF clones may harbor severe conformational or functional defects. Relying solely on VAF cannot accurately assess the therapeutic potential of reactivators (56, 60).

### 3.4 Complex protein interaction networks further elude VAF-based assessment

Beyond mutation type itself, the biological consequences of TP53 mutations are also shaped by context-dependent protein interaction networks (61). Different TP53 mutants exhibit distinct interaction profiles with p53 family members, molecular chaperones, and signaling regulators, resulting in heterogeneous downstream functional outputs (62).

Importantly, these interaction states are dynamic and may change during disease evolution or therapeutic exposure (63). For example, stabilization of mutant p53 by HSP90 can enhance dominant-negative or gain-of-function activities without any change in TP53 VAF (61). Similarly, mutant-specific interactions with p63/p73 may produce distinct apoptotic phenotypes despite identical mutation burdens (62).

Because VAF measures only the proportion of mutant alleles, it provides no information regarding protein conformation, interaction networks, or functional state (50). Incorporating functional characterization of mutant-specific protein interaction profiles may therefore improve prediction of venetoclax response (21).

## 4 Current advances in clinical and preclinical research on venetoclax-based treatment strategies for TP53-mutated AML

### 4.1 Limitations of the efficacy of hypomethylating agents combined with venetoclax in TP53-mutated AML

In TP53-mutated AML, the efficacy of venetoclax combined with HMAs is significantly limited. Mechanistically, venetoclax-induced apoptosis requires intact p53 function, a prerequisite disrupted by TP53 mutations, resulting in intrinsic resistance to BCL-2 inhibition (11). Beyond impairing apoptosis, TP53 mutations promote leukemia cell survival through maintenance of epichaperome network function, representing an additional resistance mechanism (32).

Preclinical models demonstrate preferential expansion of TP53-mutated clones following venetoclax monotherapy, confirming that this mutation directly drives acquired resistance (53). Clinical trial data reinforce this finding: even with intensified regimens such as DEC10-VEN (10-day decitabine combined with venetoclax), the outcome of TP53-mutated AML patients remains poor (53). The benefit is particularly limited in the context of high-risk genetic features, including complex karyotype or chromosome 7 deletion, with subgroup analyses failing to demonstrate significant clinical improvement (64).

Stratification based on TP53 mutation burden further quantifies this poor prognosis (7, 65). Using criteria such as TP53HR (≥1 mutation or VAF ≥40%), median OS in this high-risk group ranges from only 5.9months (65). Our recently published meta-analysis confirms these findings (5), reporting a pooled complete remission or Complete Remission with Incomplete hematological recovery (CR/CRi) rate of only 44% (95% CI: 0.32–0.57) in TP53-mutated patients, significantly lower than in IDH-mutated (71%) or FLT3-mutated (64%) subgroups.

These data underscore the particularly dismal prognosis of TP53-mutated AML and highlight the inability of current HMA-venetoclax combinations to overcome the synergistic negative impact of TP53 mutations and high-risk cytogenetic abnormalities (53).

### 4.2 Limited efficacy of TP53-targeted strategies as monotherapy or in combination with venetoclax strategies aimed at restoring p53 function, including MDM2 inhibitors and TP53 reactivators, have thus far demonstrated limited clinical efficacy in TP53-mutated AML

MDM2 inhibitors (e.g., idasanutlin, milademetan) are mechanistically dependent on the wild-type p53, as they function by inhibiting MDM2-mediated p53 degradation (66). In TP53-mutated AML where p53 transcriptional activity is already lost, this mechanism is rendered ineffective. A phase I study of milademetan plus low-dose cytarabine (LDAC) with or without venetoclax (NCT03634228) illustrated this limitation: even in TP53 wild-type AML, the objective response rate was only approximately 13% (2/16), accompanied by significant gastrointestinal toxicity (67). The study excluded TP53-mutated patients, indirectly underscoring the futility of this approach in populations with impaired p53 function (67).

p53 reactivators such as eprenetapopt (APR-246) are designed to directly target mutant p53 and restore its wild-type conformation (60). In a phase I/II study combining eprenetapopt with azacitidine and venetoclax, a complete remission rate of 37% was achieved in TP53-mutated AML (67). While this represents an improvement over the 17% CR rate observed with eprenetapopt plus azacitidine alone, the duration of response and median overall survival remained suboptimal, indicating that partial restoration of p53 function is insufficient to durably overcome venetoclax resistance (67).

The limited efficacy of these targeted strategies stems from multiple interconnected factors that extend beyond p53 itself. Firstly, leukemic cell differentiation state modulates drug sensitivity: monocytic AML (FAB M4/M5) exhibits intrinsic resistance to both MDM2 inhibitors and venetoclax, associated with CEBPB-mediated upregulation of MCL-1 and BCL2A1 and suppression of executioner caspases CASP3/CASP6 (68). Second, TP53-mutated cells engage immune evasion mechanisms, including downregulation of MHC class I and upregulation of PD-L1, which may limit the durability of response (69). Third, compensatory activation of alternative survival pathways—such as RAS/MAPK-mediated MCL-1 stabilization or FLT3-ITD signaling—provides escape routes when the p53 axis is targeted (70). Additionally contributing factors include genomic instability, upregulation of multidrug resistance proteins, and enhanced stem cell properties (71).

In summary, the poor response of TP53-mutated AML to current p53-targeted therapies reflects a convergence of cell-intrinsic, differentiation-dependent, microenvironmental, and compensatory survival mechanisms. Critically, these strategies have largely failed because they treat all TP53 mutations as functionally homogeneous, overlooking the mutant-specific functional heterogeneity detailed in Section 2. Overcoming this challenge therefore requires a paradigm shift from single-mechanism agents to combination strategies designed around mutant-specific vulnerabilities (72). A summary of key clinical trials investigating these p53-targeted strategies in AML is provided in **Table 1**, anchoring the clinical statements presented above. Building on these limitations, Section 5 outlines future directions toward a function-driven precision medicine framework. An important exception may be structurally targetable mutants such as Y220C, which contain mutation-specific druggable pockets and may therefore respond differently to p53 reactivation strategies (57).

**Table 1:** Clinical Efficacy of TP53-Targeted Strategies in AML.

Study	Regimen	Patient Population	CR/ORR	Toxicity	Ref.
Senapati et al.	Milademetan + LDAC ± venetoclax	TP53 wild-type AML	ORR: ~13% (2/16)	Significant GI toxicity	(66)
Garcia-Manero et al.	Eprenetapopt + azacitidine + venetoclax	TP53-mutated AML	CR: 37%	–	(67)
Sallman et al.	Eprenetapopt + azacitidine	TP53-mutated MDS/AML	CR: 17%	–	(60)

## 5 Future research directions and proposed solutions

The preceding sections have established that TP53 mutations mediate venetoclax resistance through diverse mechanisms, and that current VAF-based stratification fails to capture the functional heterogeneity underlying distinct resistance phenotypes (73). Translating these insights into clinical benefit will require a concerted research effort to bridge substantial knowledge gaps. The following directions represent anticipated priorities based on current understanding, though considerable work remains to elucidate how different TP53 mutants differentially modulate venetoclax sensitivity and to translate these insights into clinical practice.

### 5.1 Establishing a precision treatment system based on functional classification of mutants

TP53 mutations in AML exhibit high functional heterogeneity, with different mutant subtypes showing significant differences in their impact on apoptotic pathways and, potentially, in sensitivity to BCL-2 inhibitors (21). This functional diversity calls for a precision treatment system that goes beyond traditional VAF-based stratification. Achieving this will require several lines of investigation.

First, high-throughput functional screening technologies, including deep mutational scanning and drug sensitivity platforms, will be essential for systematically constructing a landscape of TP53 mutants, clarifying the response characteristics of each mutant subtype to venetoclax (32). The feasibility of this approach has already been demonstrated by systematic p53 mutational scanning, which generated a functional landscape of thousands of p53 DNA-binding domain variants (51). Similar strategies, when integrated with venetoclax sensitivity assays and AML-specific cellular models, could help define mutant-specific resistance phenotypes and guide function-based therapeutic stratification. Second, CRISPR/Cas9-engineered isogenic cell models can enable dynamic analysis of the clonal evolution patterns and resistance mechanisms under drug selection pressure for different mutants (74). Third, biomarkers capable of reflecting mutant functional status, rather than merely their presence, must be identified to enable molecular subtype-guided individualized treatment (75). An important consideration is that some mutants may retain partial wild-type p53 function, and such patients may still exhibit certain sensitivity to venetoclax-based combination therapies (47). However, building the comprehensive genotype-phenotype database required to support such stratification remains a formidable undertaking.

### 5.2 Developing novel multi-omics-driven biomarkers

Overcoming resistance in TP53-mutated AML will require biomarker system capable of accurately predicting treatment response (7, 76). Existing evidence indicates that TP53 mutation status or VAF cannot reliably predict the efficacy of venetoclax, underscoring the urgent need for integrated multi-omics assessment (50).

Genomic analyses have revealed that co-occurring mutations can further impair sensitivity (77). Transcriptomic studies have identified monocytic differentiation phenotype as significantly associated with intrinsic resistance (78). Metabolomic profiling has highlighted dysregulated mitochondrial dynamics as an important resistance driver (79). Future efforts should be dedicated to constructing multidimensional predictive models that integrate genomic alterations, transcriptional programs, epigenetic features, and microenvironment composition (76). Importantly, the dynamic evolution of clonal architecture during treatment may hold greater predictive value than baseline testing, necessitating real-time monitoring approaches such as liquid biopsy-based minimal residual disease (MRD) assessment (80).

### 5.3 Mutation-specific therapeutic vulnerabilities: lessons from Y220C

TP53 Y220C provides one of the strongest proof-of-concept examples supporting the transition from mutation-based classification to function-guided precision therapy (57).

Among TP53 hotspot mutations, Y220C represents a unique and clinically relevant example of mutation-specific druggability. Unlike most TP53 mutants, the Y220C substitution creates a solvent-accessible hydrophobic cavity within the DNA-binding domain, resulting in reduced protein stability while preserving partial structural integrity and residual function (58). This mutation-induced pocket provides a unique opportunity for direct pharmacological targeting.

The discovery of this structural vulnerability has enabled the development of several mutant-specific stabilizers, encompassing both covalent fragments targeting the mutant Cys220 residue (81) and the first-in-class, non-covalent Y220C reactivator rezatapopt (PC14586) (57). By binding the mutation-induced cavity, these compounds restore wild-type-like protein stability and partially recover p53 tumor suppressor activity (57). Recent studies further suggest that Y220C reactivation involves not only structural stabilization but also restoration of long-range allosteric communication and DNA-binding competence (82). In addition, cavity-directed strategies may extend to related variants such as Y220N and Y220S (83), while alternative approaches, including mutant-selective degradation and AI-guided drug discovery, are expanding the therapeutic landscape (84).

Collectively, Y220C provides compelling evidence that individual TP53 mutations possess distinct structural and functional vulnerabilities that cannot be captured by VAF alone. The successful development of Y220C-targeted therapies supports a shift from mutation burden-based classification toward function-guided precision therapeutic strategies in TP53-mutated AML (47).

Whether restoration of Y220C structural stability by rezatapopt is sufficient to re-establish non-transcriptional interactions between p53 and BCL-2 family proteins remains unknown and warrants further investigation (85).

### 5.4 Developing combination strategies targeting mutant-specific properties

Given the heterogeneous nature of TP53-mutated AML, the core of future treatment strategies lies in shifting from “targeting a single gene” to “targeting specific functional mutants” (32). This requires classifying mutants based on their key biological roles in leukemogenesis and progression, and accordingly designing precise combination regimens (47).

First, targeting should be stratified based on the functional impact of the mutant. Different TP53 mutants exhibit significant differences in protein stability, transcriptional activity, and protein interaction networks, leading to distinct pathogenic mechanisms and resistance phenotypes (21). Systematically functionally classifying common mutants to identify their respective “druggable vulnerabilities” is a key priority (57).

Second, intervention should focus on common downstream hub signaling nodes. Although upstream events differ among mutants, they may drive malignant progression and therapy resistance by converging on common downstream signaling molecules (32). Identifying these key signaling nodes and transcription factors coordinately activated by mutants and developing corresponding inhibitors holds promise for blocking aberrant signal transduction and reversing resistant (86).

## 6 Conclusion

Venetoclax resistance in TP53-mutated AML is driven by multiple interconnected mechanisms, including apoptotic dysregulation, metabolic reprogramming, leukemia stem cell expansion, and epigenetic alterations. The current VAF-based evaluation system fails to capture the functional heterogeneity of TP53 mutants that underlies diverse resistance phenotypes, and existing targeted strategies have shown limited clinical efficacy. Future progress requires a shift toward function-driven precision medicine: integrating multi-omics data to establish mutant functional classification, developing dynamic biomarkers, and designing genotype-informed combination therapies. Such approaches hold promise for overcoming resistance and improving outcomes in this high-risk population.
